# Novel Radiographic Indexes for Elbow Stability Assessment: Part B—Preliminary Clinical Study

**DOI:** 10.1007/s43465-021-00399-1

**Published:** 2021-04-28

**Authors:** Francesco Luceri, Davide Cucchi, Enrico Rosagrata, Carlo Eugenio Zaolino, Alessandra Menon, Mattia Radici, Andrea Zagarella, Michele Catapano, Mauro Battista Gallazzi, Paolo Angelo Arrigoni, Pietro Simone Randelli

**Affiliations:** 1 U.O.C. Clinica Ortopedica e Traumatologica Universitaria CTO, Azienda Socio Sanitaria Territoriale Centro Specialistico Ortopedico Traumatologico Gaetano Pini-CTO, Piazza Cardinal Ferrari 1, 20122 Milan, Italy; 2 Department of Orthopaedics and Trauma Surgery, Universitätsklinikum Bonn, Venurberg-Campus 1, 53127 Bonn, Germany; 3 U.O.C. 1° Clinica Ortopedica, Azienda Socio Sanitaria Territoriale Centro Specialistico Ortopedico Traumatologico Gaetano Pini-CTO, Piazza Cardinal Ferrari 1, 20122 Milan, Italy; 4 Laboratory of Applied Biomechanics, Department of Biomedical Sciences for Health, University of Milan, Via Mangiagalli 31, 20133 Milan, Italy; 5 REsearch Center for Adult and Pediatric Rheumatic Diseases (RECAP-RD), Department of Biomedical Sciences for Health, University of Milan, Via Mangiagalli 31, 20133 Milan, Italy; 6 Residency Program, University of Milan, Via Mangiagalli 31, 20133 Milan, Italy; 7 Servizio di Radiologia, Azienda Socio Sanitaria Territoriale Centro Specialistico Ortopedico Traumatologico Gaetano Pini-CTO, Milan, Italy

**Keywords:** Coronoid process, Elbow instability, Proximal ulna, Opening angle, Congruency, Olecranon, Radiographic study

## Abstract

**Introduction:**

The coronoid process plays a key-role in preserving elbow stability. Currently, there are no radiographic indexes conceived to assess the intrinsic elbow stability and the joint congruency. The aim of this study is to present new radiological parameters, which will help assess the intrinsic stability of the ulnohumeral joint and to define normal values of these indexes in a normal, healthy population.

**Methods:**

Four independent observers (two orthopaedic surgeons and two radiologists) selected lateral view X-rays of subjects with no history of upper limb disease or surgery. The following radiographic indexes were defined: trochlear depth index (TDI); anterior coverage index (ACI); posterior coverage index (PCI); olecranon–coronoid angle (OCA); radiographic coverage angle (RCA). Inter-observer and intra-observer reproducibility were assessed for each index.

**Results:**

126 subjects were included. Standardized lateral elbow radiographs (62 left and 64 right elbows) were obtained and analysed. The mean TDI was 0.46 ± 0.06 (0.3–1.6), the mean ACI was 2.0 ± 0.2 (1.6–3.1) and the mean PCI was 1.3 ± 0.1 (1.0–1.9). The mean RCA was 179.6 ± 8.3° (normalized RCA: 49.9 ± 2.3%) and the mean OCA was 24.6 ± 3.7°. The indexes had a high-grade of inter-observer and intra-observer reliability for each of the four observers. Significantly higher values were found for males for TDI, ACI, PCI and RCA.

**Conclusion:**

The novel radiological parameters described are simple, reliable and easily reproducible. These features make them a promising tool for radiographic evaluation both for orthopaedic surgeons and for radiologists in the emergency department setting or during outpatient services.

**Level of evidence:**

Basic Science Study (Case Series).

**Clinical relevance:**

The novel radiological parameters described are reliable, easily reproducible and become handy for orthopaedic surgeons as well as radiologists in daily clinical practice.

## Introduction

The elbow joint is a complex hinged joint that includes the distal end of the humerus in the upper arm and the proximal ends of the ulna and radius in the [1, 2]. The combination of an osseous buttresses and a soft-tissue envelope provides static and dynamic stability to the joint. Elbow stabilizers are classified into primary and secondary depending on their relative contribution to joint stability [3, 4].

Among the bony constraints, the ulnohumeral joint plays a primary stabilizing role, whereas the radio-humeral joint has only a secondary function. The biomechanical role of the coronoid process, which acts mainly as anterior support against posterior displacement of the ulna and forearm, has been thoroughly studied [4–7].

The role of the anatomical congruency between the greater sigmoid notch (GSN) and the humeral trochlea has been established by assessing specific contribution of olecranon and coronoid process to stability. A linear correlation between progressive olecranon resection and varus–valgus and rotational instability was reported [8]. The height of the coronoid process plays a key-role against posterior, rotational and varus–valgus laxity and a bone loss of more than 50% is associated to major elbow instability [5, 9].

Regan and Morrey stratified the coronoid fractures based on the percentage of coronoid involvement [10]: Type I describes an avulsion of the coronoid tip. Type II indicates a single or comminuted fragment involving less than 50% of the coronoid; when the fracture involves more than 50% of the coronoid process it is classified as a type III fracture. A modification was later added for fractures without dislocation (A), and fractures with an associated dislocation (B).

 The Regan and Morrey classification system is widely used, but a limitation of this classification system, already described [11], is the lack of a specific thresholds to define Types I and II fractures with potential overlap between the two types. This classification does not fully describe the progressive loss of joint congruence in a fracture setting and does not give any precise information about the intrinsic stability of the elbow, especially in those cases in which the clinicians do not have pre-trauma radiographs available.

Currently, we have a huge theoretical knowledge of the anatomy of the GSN, but simple radiological tools to evaluate the intrinsic elbow stability in a clinical setting are lacking. The plain radiography is the basic tool to study elbow joint. Practical and reliable radiographic indexes, able to describe the ulnar congruency, could be helpful in regular clinical practice and in the emergency service.

The aim of this study was to introduce new radiological indexes for lateral plain radiographs that may help quantify the functional anatomy of the elbow joint and to evaluate their inter-observer and intra-observer reproducibility. The secondary goal of this study was to define the normal values of these indexes in a healthy population without the history of trauma or instability.

## Materials and Methods

### Patients

All elbow radiographs performed on patients younger than 18 years and older than 75 years referring to the Emergency Department of our Institution for elbow pain of any origin during 2018 (12 months) were considered for inclusion in the study. To select only radiographs without evidence of any elbow pathology, the clinical records were reviewed to exclude patients with local or systemic bone or joint disease, history of trauma or signs of instability at the clinical evaluation. Radiographs were considered eligible for further evaluation if the criteria reported were fulfilled:

### Inclusion Criteria

1. Skeletally mature subjects aged between 18 and 75 years old
2. Standard lateral elbow radiographs, which fulfilled the following quality criteria:
3. 90° elbow flexion with concentric trochlear sulcus contour [12]

### Exclusion Criteria

1. Congenital or developmental anomaly of elbow, arm or forearm
2. Systemic disease/local pathological abnormality of the bony anatomy
3. History of previous elbow fracture or dislocation
4. Elbow osteoarthritis
5. History of elbow surgery
6. Radiographs not fulfilling aforementioned quality criteria

### Radiological Evaluation

**Fig. 1:**
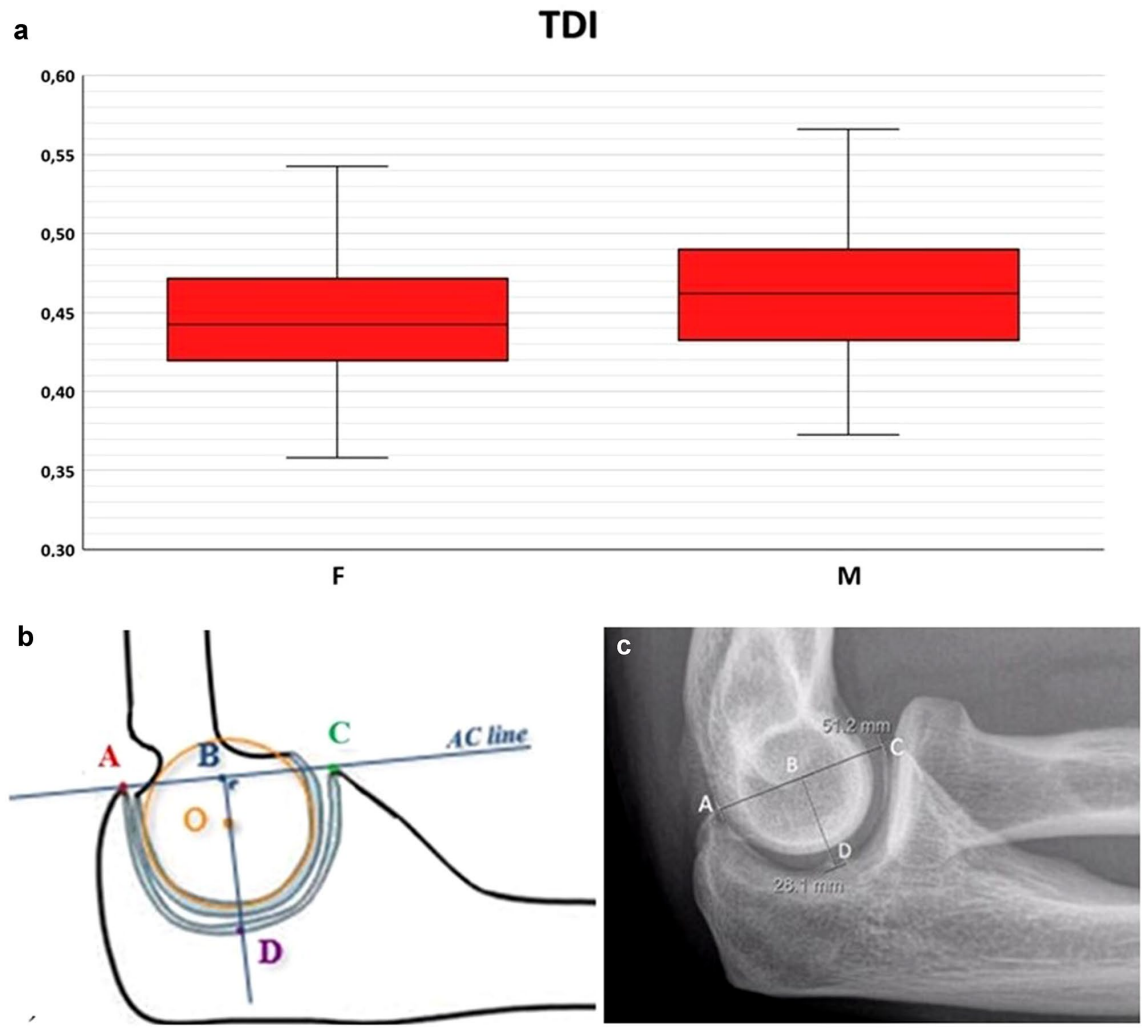
Box-and-whiskers plot (**a**) illustrating the trochlear depth index (TDI) comparison between males and females (*p* < 0.05). Schematic representation (**b**) and measurement on a lateral radiograph (**c**) of TDI: the ratio between the distance from the olecranon tip to the coronoid tip (AC) and the distance between this line and the deepest point of the trochlea (TDI = BD/AC)

**Fig. 2:**
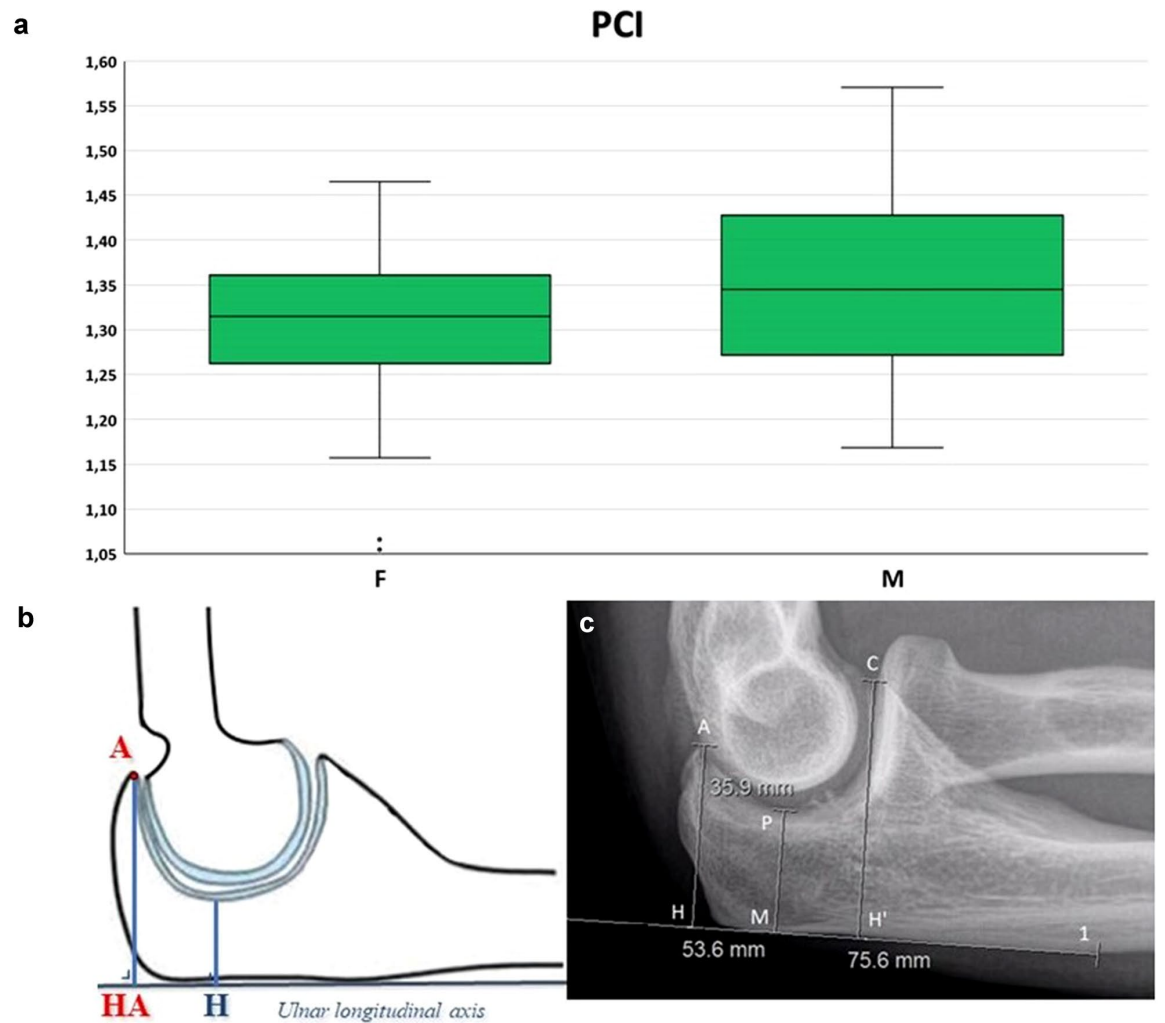
Box-and-whiskers plot (**a**) illustrating the posterior coverage index (PCI) comparison between males and females (*p* < 0.05). Schematic representation (**b**) and measurement on a lateral radiograph (**c**) of PCI: the ratio between the olecranon height (HA, measured as the shortest distance from a line on the posterior ulnar surface to the olecranon tip) and the minimal trochlear height (H)

**Fig. 3:**
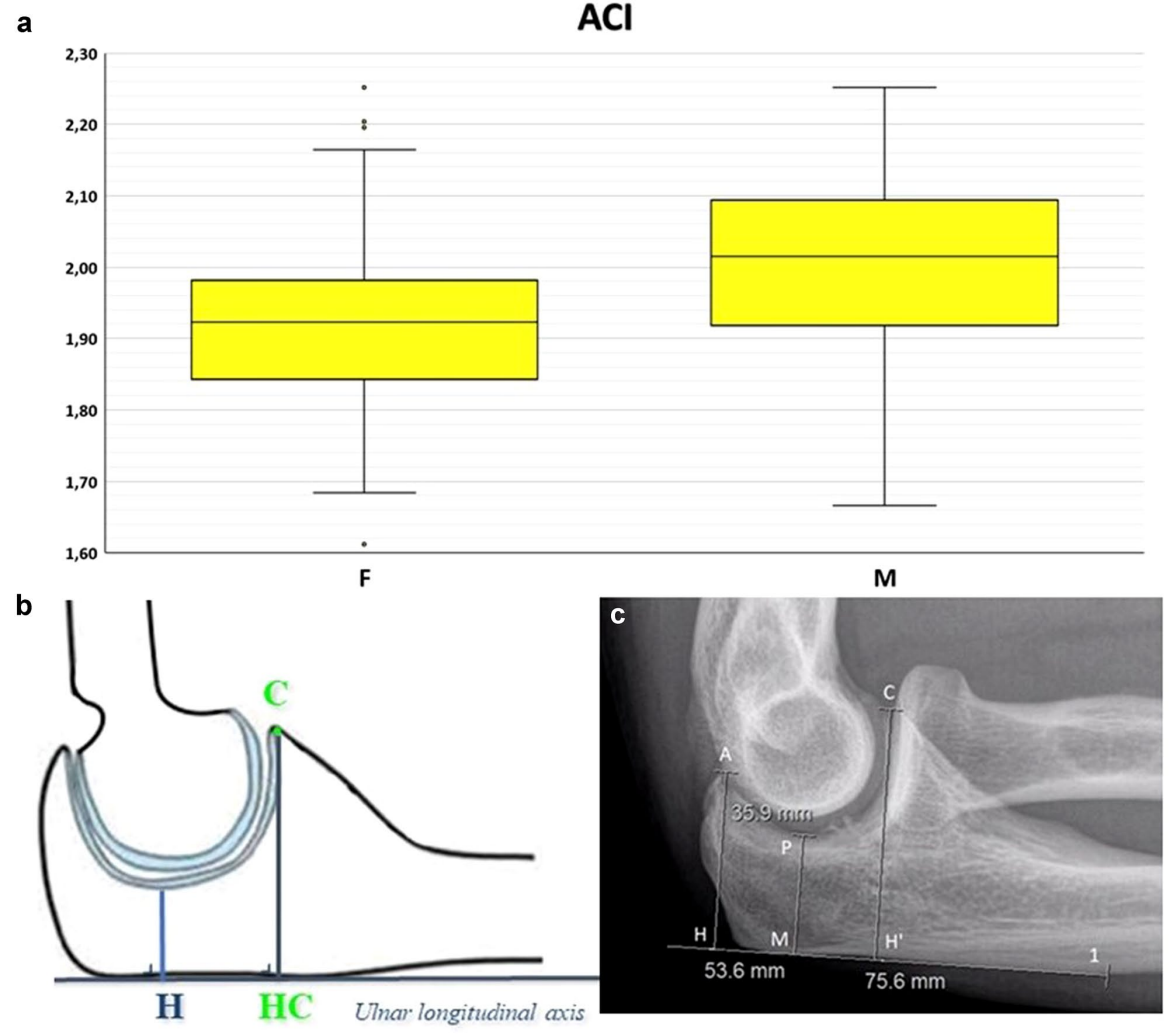
Box-and-whiskers plot (**a**) illustrating the anterior coverage index (ACI) comparison between males and females (*p* < 0.05). Schematic representation (**b**) and measurement on a lateral radiograph (**c**) of ACI: the ratio between the coronoid height (HC, measured as the shortest distance from a line on the posterior ulnar surface to the coronoid tip) and the minimal trochlear height (H)

**Fig. 4:**
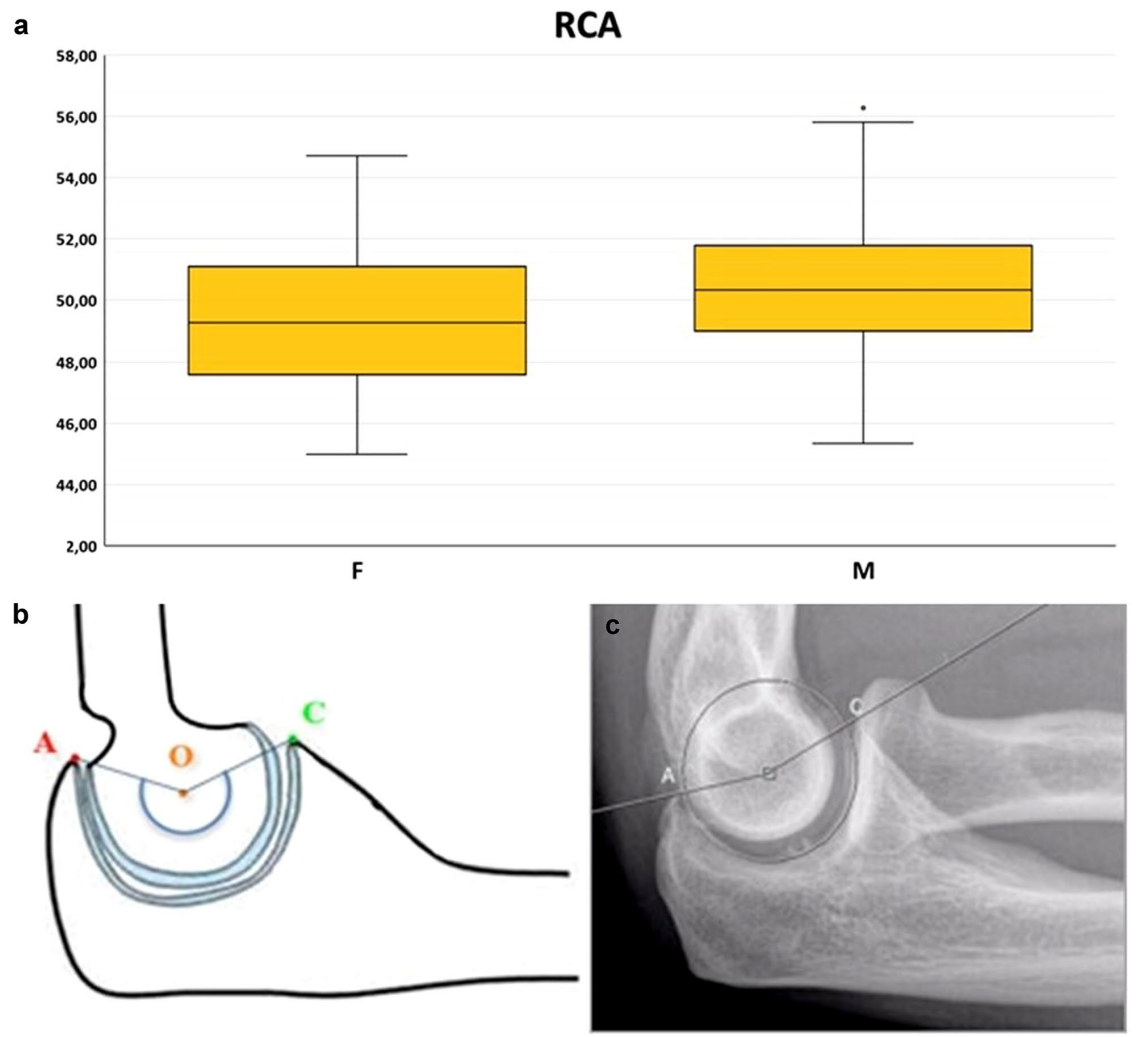
Box-and-whiskers plot (**a**) illustrating the radiographic coverage angle (RCA) comparison between males and females (*p* < 0.05). Schematic representation (**b**) and measurement on a lateral radiograph (**c**) of the RCA, defined as the dorsally opened angle between a line passing through the centre of a circle tangent to GSN surface and the olecranon tip and a line passing through the centre of the same circle and the coronoid tip (i.e. the angle subtended by the circular segment AOC). Alternatively, the RCA can be alternatively derived by mathematical operations form the linear measurements illustrated in Fig. 1 as *RCA* = *4 ∙ arctang (2 ∙ BD / AC)*

**Fig. 5:**
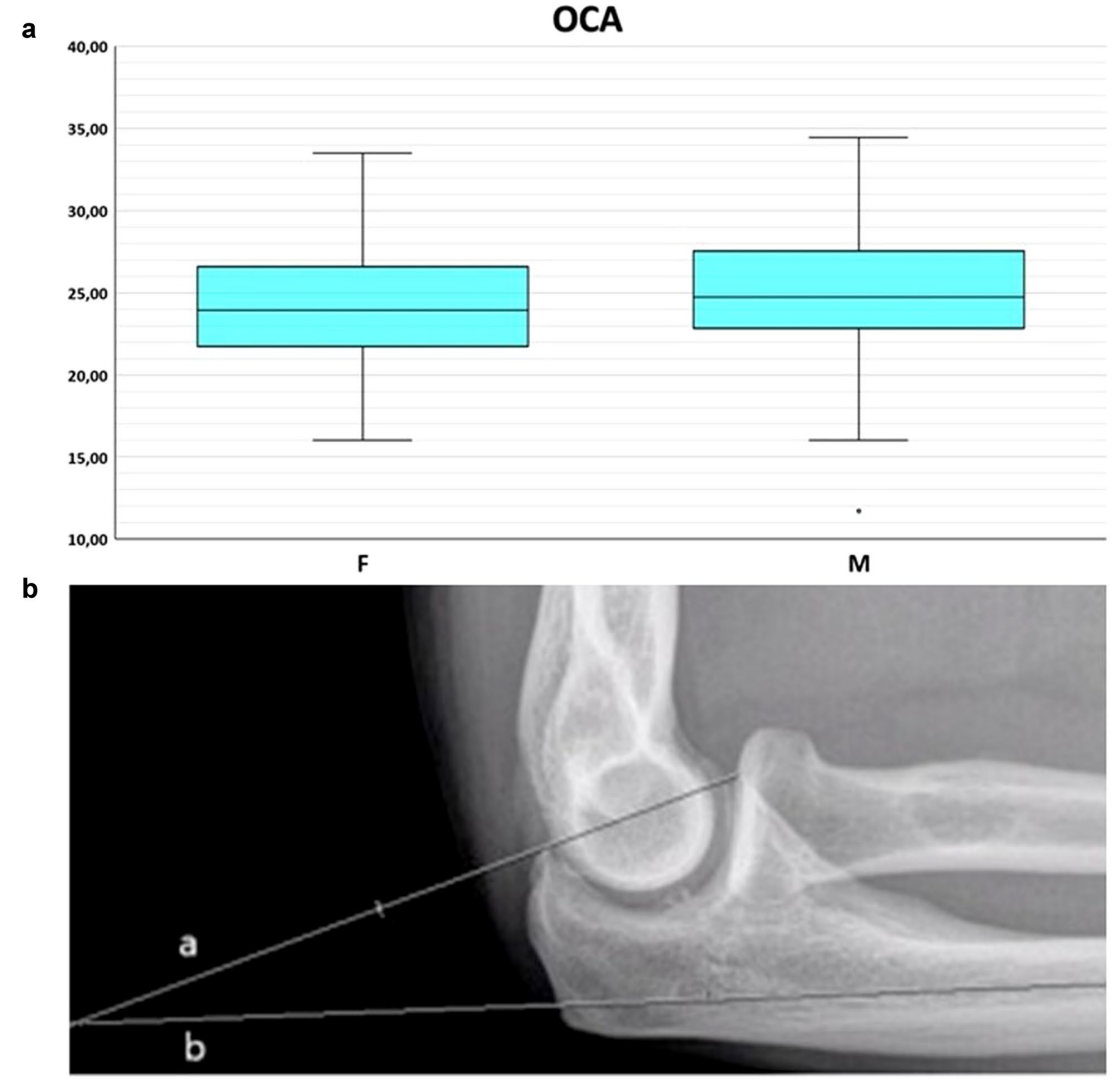
Box-and-whiskers plot (**a**) illustrating the olecranon–coronoid angle (OCA) comparison between males and females (*p* < 0.05). OCA: angle between the line passing through coronoid and olecranon tips (**a**) and the ulnar diaphysis (**b**) [13]

On each included lateral radiograph, the tip of the olecranon (A), the tip of the coronoid tip (B), the center (O) of the GSN and its deepest point (D) as well as a line tangent to the posterior cortex surface of the proximal ulna and the ulnar diaphysis were identified as radiographic landmarks. These landmarks were used to define three primary indexes and two angles (Fig. 1–5): trochlear depth index—TDI; posterior coverage index—PCI; anterior coverage index—ACI; radiographic coverage angle—RCA and olecranon-coronoid angle—OCA [13]. The RCA was then normalized to the value of 360°, representing the whole circumference of the GSN.

A digital software (IMPAX Agfa HealthCare) was used to mark all radiographic landmarks and to generate semiautomatically linear and angular measures. All investigated indexes and angles are not affected by the bias linked to the radiographic projection magnification.

### Examiners

All radiographs were evaluated and measured by four independent observers with extensive experience in the field of musculoskeletal radiology or orthopaedic surgery (more than 10 years): two of them were dedicated musculoskeletal radiologists (Examiner 1 and Examiner 2) and two of them were orthopaedic surgeons (Examiner 3 and Examiner 4).

All four observers evaluated each X-ray twice, with a 15-day delay between first and second assessments to perform an *internal validation*.

The University Hospital Centre Review Board approved the study protocol (*Comitato Etico Milano Area 2, 595_2019bis*). All procedures performed in studies involving human participants were in accordance with the ethical standards of the institutional and/or national research committee and with the 1964 Helsinki Declaration and its later amendments or comparable ethical standards.

### Statistical Analysis

Statistical analysis was performed by one investigator (A.M.) using GraphPad Prism v 6.0 software (GraphPad Software Inc) and with SPSS v.15.0 (SPSS Inc., an IBM Company, Chicago, IL, USA). The Shapiro–Wilk normality test was used to evaluate the distribution of the sample [14]. Continuous variables were expressed as mean ± standard deviation (SD) or as median and first and third quartiles [Q1–Q3] as, as appropriate; dichotomous variables were expressed in numbers of cases and frequencies.

The inter-observer reproducibility of the radiographic indexes was evaluated with intra-class correlation coefficient (ICC), which were derived from one-way random-effect analysis of variance. Intra-observer ICC estimates were calculated based on the single measurements, using an absolute-agreement, two-way mixed-effects model. Inter-observer ICC estimates were calculated based on the mean value between the two measurements of each observer. The ICC was considered moderate if between 0.500 and 0.749, good if between 0.750 and 0.899 and good and excellent if ICC > 0.90.

Correlations between different measurements were investigated using Pearson coefficient (“*r*”) for normally distributed variables and Spearman coefficient (“*ρ*”) for non-normally distributed variables. The same parameters were used to evaluate correlation between measurements and age of the subjects. Correlations between indexes and categorical variables (side, sex) were evaluated using Student’s *t* test for normally distributed indexes, Mann–Whitney test was used for non-normally distributed variables [15]. For all analyses, the significance level was set at P value lower than 0.05.

## Results

237 consecutive standard lateral radiographs were screened for eligibility. 126 lateral radiographs of the elbow (62 left and 64 right elbows) fulfilled the inclusion criteria and were analysed.

The study population encompassed 126 subjects (62 females and 64 males), with a mean age of 44.9 ± 15 years (18–75 years).

The mean TDI was 0.46 ± 0.06 (0.3–1.6); the mean ACI was 2.0 ± 0.2 (1.6–1) and the mean PCI was 1.3 ± 0.1 (1.0–1.9). The mean RCA was 179.6 ± 8.3° (normalized RCA: 49.9 ± 2.3%) and the mean OCA was 24.6 ± 3.7°.

With the sole exception of the RCA, all indexes had good inter-observer and intra-observer reliability. Inter-observer ICC was 0.727 (0.641–0.797) for TDI; 0.709 (0.616–0.783) for ACI; 0.724 (0.636–0.795) for PCI, 0.522 (0.372–0.643) for RCA and 0.843 (95% CI 0.679–0.912) for OCA (Table 1).

**Table 1:** Inter-observer reliability of the investigated parameters

Inter-observer	ICC	95% IC
TDI	0.727	0.641–0.797
ACI	0.709	0.616–0.783
PCI	0.724	0.636–0.795
RCA	0.522	0.372–0.643
OCA	0.843	0.679–0.912

Intra-observer reliability values for the four observers were also moderate to excellent (Table 2).

**Table 2:** Intra-observer reliability of the investigated parameters

Intra-observer	ICC	95% IC
TDI1	0.792	0.717–0.849
TDI2	0.818	0.749–0.869
TDI3	0.532	0.394–0.646
TDI4	0.833	0.758–0.884
ACI1	0.717	0.620–0.793
ACI2	0.807	0.737–0.861
ACI3	0.620	0.499–0.716
ACI4	0.841	0.776–0.888
PCI1	0.597	0.471–0.699
PCI2	0.899	0.860–0.928
PCI3	0.487	0.343–0.609
PCI4	0.782	0.703–0.841
RCA1	0.630	0.512–0.724
RCA2	0.933	0.907–0.953
RCA3	0.613	0.491–0.711
RCA4	0.795	0.720–0.851
OCA1	0.712	0.612–0.789
OCA2	0.911	0.876–0.937
OCA3	0.867	0.816–0.905
OCA4	0.943	0.907–0.963

Observers: two dedicated musculoskeletal radiologists (Examiner 1 and Examiner 2) and two orthopaedic surgeons (Examiner 3 and Examiner 4)

No significant correlation was found between the radiological indexes and the age of the patients. There was statistically significant difference between males and females in terms of TDI, ACI, PCI and normalized RCA but not OCA (Figs. 1, 2, 3, 4, 5); Higher values for all indexes were reported for males (TDI: males: 0.47 ± 0.06; females: 0.45 ± 0.06; *p* = 0.047—ACI: median in males: 2.09 [1.92–2.09]; median in females: 1.92 [1.84–1.98], *p* < 0.001—PCI: median in males: 1,35 [1.27–1.43]; median in females: 1.31 [1.26–1.36], *p* = 0.029—RCA: males: 50.35% ± 2.2%; females: 49.4% ± 2.3% *p* = 0.022).

**Fig. 6:**
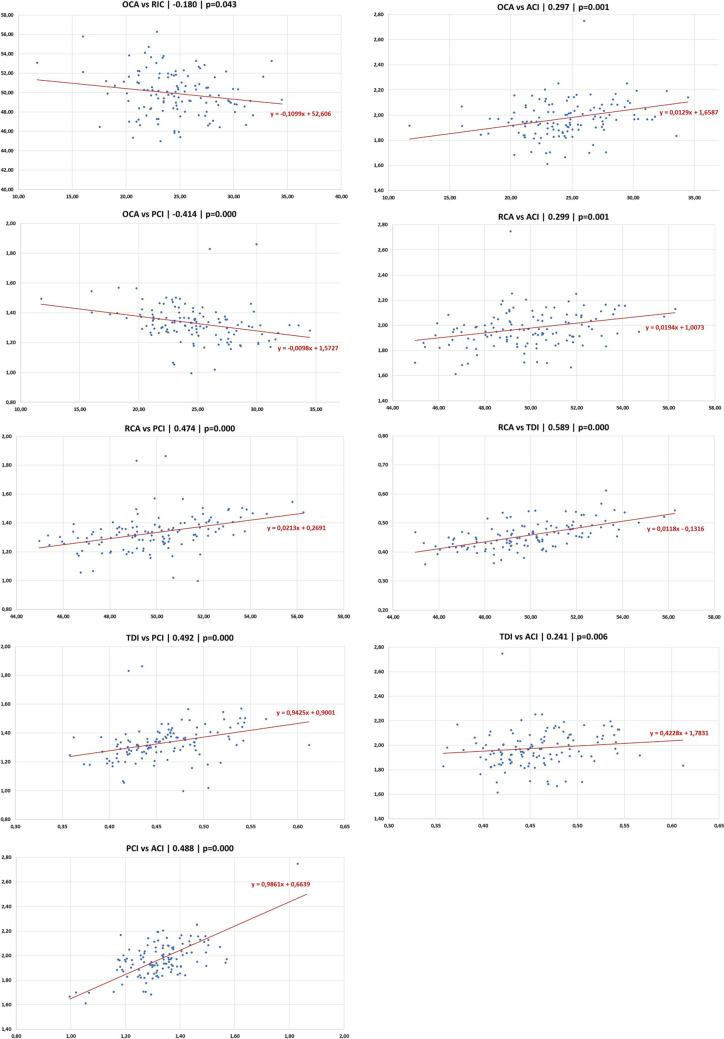
Dispersion plots showing correlations between radiological indexes (*: *p* < 0.01)

Several correlations were measured between the different indexes. Strength of correlation was moderate for OCA–PCI, RCA–TDI, RCA–PCI, TDI–PCI and ACI–PCI and weak for OCA–ACI, RCA-ACI, TDI-ACI and OCA-RCA, thus excluding redundancy between the evaluated indexes (Fig. 6). Interestingly, the strength of the correlation between the measured RCA and TDI, which are geometrically linked by the equation *RCA* = *4* ∙ *arctang* *(2* ∙ *TDI)* was by far lower than expected (r = 1), herewith suggesting low reliability of the angular measurement RCA.

## Discussion

The main finding of this study is that all radiological indexes and angles investigated, with the exception of the RCA, have a good inter- and intra-observer reliability when measured in a healthy population.

A tendency for a more congruent anatomy was observed in males as compared to females.

No relevant redundancy between the parameters TDI, ACI, PCI and OCA was reported, with only moderate or weak correlations existing between them. This study revealed also that an angular measurement of the GSN coverage is less reproducible than linear measurements, therefore, encouraging the use of the latter, which also permits to calculate the RCA angle through mathematical operations.

TDI may be useful to quantify the anatomical congruency of the elbow joint and to highlight possible elbow instability predisposition in case of trauma setting. ACI and PCI may be extremely helpful in clinical practice to define the stabilizing role of the anterior and posterior walls. These parameters may also help the clinicians to better define different patients’ subgroups according to the increasing degree of expected elbow stability.

The anatomy of the proximal ulna has been widely studied in the setting of reconstructive as well as replacement surgery [16–18]. Simple radiological landmarks, such as the posterior cortex of proximal ulna and tips of olecranon and coronoid processes, have been used to better describe the elbow anatomy in its complexity [13, 17].

Despite the elbow joint being the second most commonly dislocated joint in adult, the exact mechanism that can cause this event with or without fracture is nowadays still a subject of debate and no consensus has been reached on the reason why some elbows experience a simple and other a complex dislocation [19].

O'Driscoll et al. [4] described a sequential soft-tissue disruption starting from the lateral side, whereas other more recent studies proposed that the soft-tissue injury could begin from the medial side [20].

Micro-trauma is another clinical scenario that can lead to symptomatic chronic instability, both affecting the medial and the lateral side. Patient with symptomatic minor instabilities complain of chronic elbow pain and limitation in daily activities, which makes demanding and time consuming the process necessary to reach a proper diagnosis and indicated adequate treatment [21–23].

These numerous pathological entities and clinical variables, in addition to the complexity of functional elbow anatomy, make it extremely challenging to identify and quantify intrinsic elbow stability with simplified imaging parameters.

Regan and Morrey stratified the coronoid fractures based on the percentage of coronoid involvement and created a classification system, which is still currently widely used [10]. Herewith, contribution of the coronoid process against posterior, rotational and varus–valgus laxity could be quantified, suggesting that more than 50% height loss is associated with major elbow instability [5, 9].

Nevertheless, static radiographic parameters have inherent limitations, as they cannot fully describe the complex joint stability status: for example, the rotation axis of the elbow is not a static constant, as it significantly shifts throughout the range of motion of the joint. The common limitation of radiographic classifications is its inability to accurately describe the three-dimensional fracture pattern and help in surgical planning. To overcome these limitations, O’Driscoll et al. [24] proposed a CT classification system for coronoid fractures, which provides also indications for the surgical decision-making [18, 25].

Although the role of the coronoid process is elbow stability indisputable, a further aspect to be considered is role of the olecranon height in generating the anatomical congruency between the GSN and the humeral trochlea: progressive olecranon resection has been correlated with increasing varus–valgus and rotational instability, suggesting that olecranon and coronoid processes both play a relevant role in determining elbow stability [8]. This raised interest in creating indexes for the olecranon height and in measuring the proximal ulna and the GSN as a whole, and several radiographic parameters were reported in adolescent and young adults [13, 26, 27].

The trochlear depth index used in this study was inspired by the acetabular depth index (depth–width ratio) and its importance in defining the joint stability for developmental dysplasia hip [28, 29]. This matches width and depth of the trochlear notch, giving a concise description of ulnar elbow coverage. Ndou and Scheparz [30] described GSN width using the same landmarks, measuring the depth as the distance from the deepest point of GSN to a line connecting olecranon and coronoid tips; however, to our knowledge, a depth–width ratio has never been proposed as a radiological parameter to assess the stability of the elbow joint. An advantage of the combination of two linear measurements as opposed to the use of an angular measurement (such as the RCA) is the lower risk of measurements errors, mainly attributable to the imprecision in determining the centre of the GSN necessary to calculate the RCA. This was confirmed in our study by the moderate ICC for the RCA measurements and the low strength of the correlation between TDI and RCA (expected *r* value = 1). The authors recommend, therefore, to rely on linear measurements and, if needed, to obtain derived angles to describe GSN coverage or sigmoid notch opening by mathematical operations, such as *RCA* = *4* ∙ *arctang (2* ∙ *BD* */* *AC)* = *4* ∙ *arctang* *(2* ∙ *TDI)* and sigmoid notch opening angle = *RCA* */* *2* [31].

Simple, practical and reliable radiographic indexes to assess elbow stability, such as those introduced in this study, may have an important role in the decision-making process, already from the first clinical consultation after injury.

These radiographic parameters are extremely promising, since they permit to obtain a practical and quick measurement of the constitutional elbow congruency, can be easily reproduced in an emergency service without the need of a CT and are applicable in both trauma and non-trauma patients.

Comparing the values obtained in the current study to previously published normal data, OCA highlights the role of the coronoid in relation to posterior dislocation. Goldfarb et al. measured a mean value of 23°, and the results are almost correlating with our results. OCA and RCA resulted to be in line with previous studies [13, 26].

ACI and PCI have never been described in the scientific literature. For coronoid, olecranon and notch height we used the same landmarks applied by Beşer et al. [17]. The strength of correlation measured between the radiographically determined ACI and PCI in the current study is similar to that obtained by Beşer et al. for the non-normalized anatomical counterparts of these two parameters measured in anatomical specimens. Interestingly, we found a moderate correlation between the PCI and OCA, whereas the mentioned anatomical study did not reveal any correlation between ulnar angles and the posterior olecranon height. These data suggest that ACI and PCI may be very helpful in clinical practice to define the stabilizing role of the anterior and posterior walls. These parameters may also help the clinicians to better define different patients’ subgroups according to the increasing degree of expected elbow stability. The hope is that these indexes will allow selecting patients with joint instability/stiffness risk factors or to better guide second level imaging after acute trauma or in post-traumatic elbow sequelae. The relatively small sample size was the most important limitations of this study. Moreover, the two-dimensional radiographic elbow images do not take into account of the complexities of bony anatomy and its fundamental role in joint stability, which was visible in some differences emerging when comparing the results with the anatomical study by Beşer et al. [17]. Finally, this radiographic study was limited to a single plane view for a primary constraint of elbow joint, and the evaluation of structures potentially affecting stability in other planes was outside the scopes of this study. CT and MRI have also been used to measure joint stability, taking into account both its bony and cartilaginous contribution; however, the high costs and limited availability of MRI in a trauma setting limit its use as a second level examination for selected patients [26]. Nevertheless, the authors encourage the use of standardized reconstructions in the sagittal plane and the measurements of the presented indexes, for whom a validation in CT and MRI is expected.

## Conclusion

Novel radiographic indexes to describe the congruency of the greater sigmoid notch and the anatomy of the proximal ulna have been evaluated in a healthy population: TDI, ACI, PCI and OCA demonstrated a good inter- and intra-observer reliability and showed no relevant redundancy. A tendency for a more congruent anatomy was observed in males as compared to females. These parameters can be used to simply and effectively describe elbow joint functional bony anatomy; on the other hand, an angular measurement of the GSN coverage proved to be less reproducible than linear measurements.

## Abbreviations

GSN: Greater sigmoid notch
TDI: Trochlear depth index
PCI: Posterior coverage index
ACI: Anterior coverage index
RCA: Radiographic coverage angle
OCA: Olecranon–coronoid angle
SD: Standard deviation
ICC: Intra-class correlation coefficient
